# Acute Effects and the Dreamy State Evoked by Deep Brain Electrical Stimulation of the Amygdala: Associations of the Amygdala in Human Dreaming, Consciousness, Emotions, and Creativity

**DOI:** 10.3389/fnhum.2020.00061

**Published:** 2020-02-25

**Authors:** George Lai, Jean-Philippe Langevin, Ralph J. Koek, Scott E. Krahl, Ausaf A. Bari, James W. Y. Chen

**Affiliations:** ^1^Neurology Service, VA Greater Los Angeles Healthcare System, Los Angeles, CA, United States; ^2^Department of Neurology, University of California, Los Angeles, Los Angeles, CA, United States; ^3^Neurosurgery Service, VA Greater Los Angeles Healthcare System, Los Angeles, CA, United States; ^4^Department of Neurosurgery, University of California, Los Angeles, Los Angeles, CA, United States; ^5^Psychiatry and Mental Health Service, VA Greater Los Angeles Healthcare System, Los Angeles, CA, United States; ^6^Department of Psychiatry and Behavior Sciences, University of California, Los Angeles, Los Angeles, CA, United States; ^7^Research and Development, VA Greater Los Angeles Healthcare System, Los Angeles, CA, United States

**Keywords:** DBS, dreamy state, double consciousness, dreaming, emotion, creativity, amygdala, PTSD

## Abstract

Accurate localization of complex human experiences such as emotions, dreaming, creativity, and consciousness to specific cerebral structures or neural networks has remained elusive despite technological advances. We report the use of acute deep brain stimulation (DBS) to evoke behavioral and emotional effects by applying electrical stimulation (ES) at various voltage strengths to the basolateral and central subnuclei of the amygdala in addition to the head of hippocampus (HC) for two subjects with medically refractory post-traumatic stress disorder (PTSD). Our results suggest that the amygdala could be a node in a neural network responsible for the generation of complex vivid mental imagery and integrated sensory experiences similar to John Hughlings Jackson’s “dreamy state” and “double consciousness,” which have been classically associated with temporal lobe epilepsy during uncinate seizures. That we were able to elicit similar vivid, dynamic, complex, bizarre, and original mental imagery with ES in non-epileptic subjects suggests that Jackson’s seizure related “dreamy state” and “double consciousness” may arise from heightened innate brain mechanisms with the amygdala acting as a node in the neural network responsible for physiologic dreaming and creative functions. Furthermore, our subjects experienced different emotions with different stimulation strengths at various electrode contacts. Our results suggest that higher voltage stimulation of the amygdala and HC at 4–5 V leads to predominantly negative responses and 2–4 V stimulation showed inversely coupled positive and negative responses of the amygdala in either hemisphere which may imply hemispheric dominance of emotional valences without relation to handedness. Due to the unique and complex responses dependent on location and strength of stimulation, we advise that all patients receiving DBS of the amygdala undergo acute stimulation mapping in a monitored setting before selecting therapeutic parameters for chronic stimulation.

## Introduction

Human brain mapping began as early as the 1800 s and was expanded by Wilder Penfield’s and Herbert Jasper’s use of cortical electrical stimulation (ES) in awake patients (Ritaccio et al., 2018). The accurate mapping of more complex human experiences, such as dreams, emotions, and creativity, however, has remained elusive. Penfield noted that “anger, joy, pleasure, sexual excitement…neither localized discharge nor ES is capable of awakening any such emotions” (Penfield and Jasper, 1954). Over time, elucidation of neuroanatomical components of such functions has progressed alongside advances in neuroimaging, polysomnography, and electrophysiology (Kussé et al., 2010). Here, we present behavioral and emotional effects evoked by acute stimulation in two subjects who underwent implantation of deep brain stimulation (DBS) electrodes within their amygdala bilaterally for the treatment of medically refractory post-traumatic stress disorder (PTSD). Amygdala ES utilizing DBS electrodes in awake subjects without epilepsy has not been previously described in the literature. Published studies are limited to depth electrode stimulation in patients with refractory epilepsy (Guillory and Bujarski, 2014). During acute amygdala ES, both of our awake subjects maintained full awareness of reality while simultaneously experiencing vivid mental imagery associated with auditory, visual, tactile, and olfactory sensations involving autobiographical or original contents with emotional valence. These phenomena were analogous to those associated with uncinate seizures described by John Hughlings Jackson as “dreamy state” and “double consciousness,” which are classically associated with temporal lobe epilepsy (TLE) (Hogan and Kaiboriboon, 2003). The reproduction of “dreamy state” and “double consciousness” by amygdala ES in non-epileptic subjects suggests that such phenomena are not only epilepsy-related. It provides an essential piece of evidence that Jackson’s “dreamy state” and “double consciousness” could arise from the over-activation (or inhibition) of an innate human brain mechanism involving the amygdala. Furthermore, the elicitation of novel, not-previously experienced mental imagery leads us to speculate that the amygdala may also be involved in creative functions. Based on our results, we hypothesize that complex human experiences such as dreams, emotions, and creativity share similar neural networks, of which the amygdala could be a node. Our findings, although limited to two case reports, may suggest a new experimental scheme for stimulating the amygdala to investigate “subject consciousness” (Taylor, 1931, 1958).

## Materials and Methods

As part of an ongoing institutional review board (IRB) approved project at the Veteran Affairs Greater Los Angeles Healthcare System (VAGLAHS) to study the utility of DBS in the treatment of refractory PTSD, two subjects had DBS electrodes (Medtronic® model 3387) and generators (Medtronic® Activa PC) implanted after obtaining informed consent. Both subjects had severe refractory PTSD for a minimum of 5 years and no remission period longer than 6 months (Koek et al., 2014). Written informed consent for this publication was obtained.

Subject 1 was a 48-year-old right-handed male combat veteran who developed debilitating medically refractory PTSD after exposure to traumatic battlefield scenes. Subject 2 was a 40-year-old right-handed male combat veteran who also developed medically refractory PTSD after suffering combat related poly-trauma. Both subjects had unremarkable brain magnetic resonance imaging (MRI) and electroencephalography (EEG) studies. Subject 1’s medications included fluoxetine, gabapentin, and prazosin. Subject 2’s medications included venlafaxine, gabapentin, buspirone, and divalproex.

**FIGURE 1:**
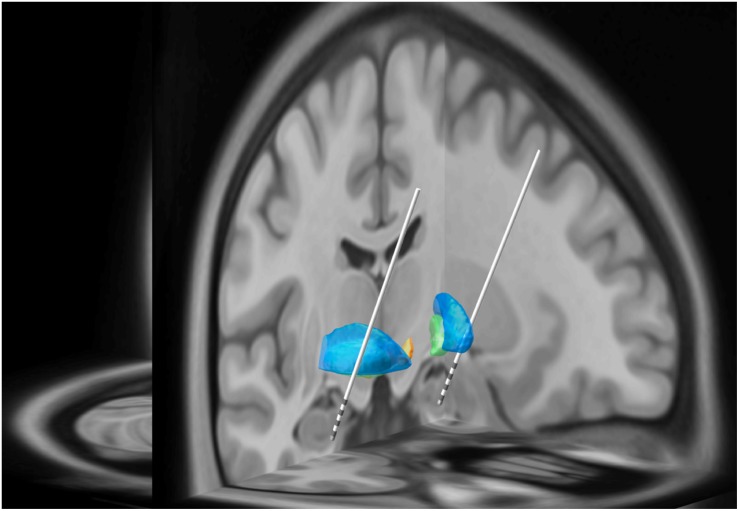
In this three-dimensional image, Subject 2’s MRI brain imaging studies were registered to the MNI space (MNI152 NLIN2009a) and then overlaid on the structural atlas of Mai (Horn and Kühn, 2015; Mai and Majtanik, 2017). Here we demonstrate the trajectory for each DBS electrode implanted into the head of the HC and amygdala as represented in “MNI space.” The blue regions represent the putamen while the green regions represent the globus pallidus. The white lines represent the DBS leads with four gray regions representing individual electrode contacts. See Figure 2 for precise localization of each contact.

**FIGURE 2:**
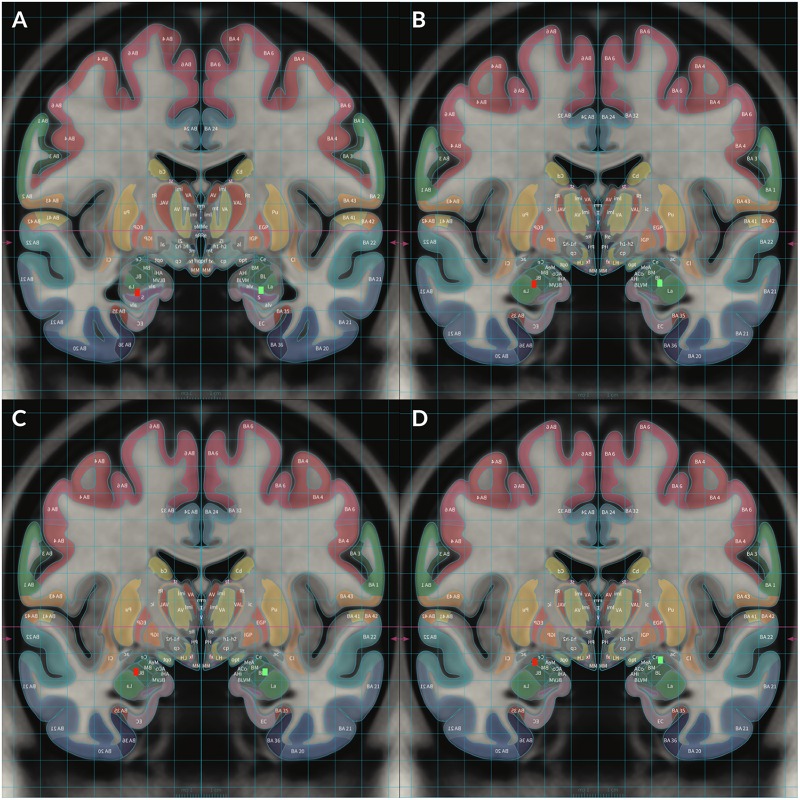
To demonstrate locations of each electrode, Subject 2’s MRI brain imaging studies were registered to the MNI space (MNI152 NLIN2009a) and then overlaid on the structural atlas of Mai (Horn and Kühn, 2015; Mai and Majtanik, 2017) to allow delineation of cerebral cortices and subcortical structures with color coded representations of cortical and subcortical parcelation according to the Mai atlas. The red rectangular markers represent right sided DBS electrode contacts 8–11 while the green rectangular markers represent left sided contacts 0–3. Contact 0 was placed in the left HC and contact 8 was placed in the right HC **(A)**. Contact 1 was placed in the left ventral BLA and contact 9 was placed in the right ventral BLA **(B)**. Contact 2 was placed in the left dorsal BLA and contact 10 was placed in the right dorsal BLA **(C)**. Contact 3 was placed in the left CeA and contact 11 was placed in the right CeA **(D)**.

Each conventional quadripolar DBS lead was implanted without complication into the amygdala bilaterally with the distal contact placed within the head of the hippocampus (HC), the two middle contacts placed within the ventral and dorsal basolateral nucleus of the amygdala (BLA), and the proximal contact placed within the central nucleus (CeA). Post-implant registration was performed to an MNI-based histological atlas to confirm that the electrodes are in the amygdala. Refer to Figures 1, 2, and a prior publication for detailed descriptions of the surgical technique and electrode positioning (Langevin et al., 2016).

One month after implantation, before initiation of chronic DBS stimulation, mapping of effects elicited by acute DBS stimulation was performed with simultaneous video EEG (VEEG) monitoring in the inpatient epilepsy monitoring unit. The subjects spontaneously provided verbal feedback describing their emotional state and overall experience (if any) throughout the stimulation procedure blinded to the change in stimulation parameters. Contacts were individually stimulated from 0–5 volt (V) in 1 V increments. The stimulation frequency and pulse-width were 160 Hz and 60 μs, respectively. For more information, refer to our prior publication of the study protocol (Koek et al., 2014). The measured impedance from the electrode contacts of both subjects ranged from 834 to 2349 Ohm, with an estimated maximal charge density of ∼12 μC/cm^2^. Charge density has to be calculated for subject safety if our experimental parameters are to be adopted using other types of electrode. Subject 1 was initially stimulated with 120 μs pulse-width at contacts 0 and 1, however, pulse width was lowered to 60 μs due to significant negative responses. Stimulation duration at each voltage strength lasted approximately 1–5 min or more dependent on responses. The contacts represented cathodes while the DBS case represented the anode. The subjects were blinded to stimulation parameters except with significant negative reactions. Repetition or cessation of stimulation was determined based upon safety considerations. The VEEG was retroactively reviewed independently by two epileptologists who categorized each subject’s reported experiences as “positive” (happiness, euphoria, relaxation, pleasure, or other positive sensations), “negative” (anxiety, anger, pain, fatigue, fear, or discomfort), or “mixed”. Responses were categorized according to emotional valence, intensity (mild/moderate vs. strong), and sensations. “Delayed” responses included symptoms occurring 1–2 min after stimulation onset. Only consensus results between the two reviewers are included. Additional symptoms were categorized as mental imagery including visual, olfactory, tactile, or auditory components. Experiences suggesting creativity were identified by the production of not-previously experienced original content.

## Results

Euphoria was evoked with 3 V right ventral BLA in Subject 1 and 2 V left ventral BLA stimulation in Subject 2. Subject 1 laughed that he “could do this all day” while reporting mental imagery of childhood locations and floating over familiar scenery. Subject 2 felt happiness, describing as “I feel some euphoria….” He felt “practically normal,” the happiest he has been since starting the telemetry session.

Both subjects experienced varying degrees of happiness. Subject 1 had more positive responses with right sided stimulation in addition to left ventral (trial 2) and dorsal BLA stimulation. Strong positive responses were reported with 4 V left ventral BLA (trial 2), 3 V right dorsal BLA, and 2 V right CeA stimulation. At other settings, he felt “happiness” while laughing at enjoyable mental imagery including a woman on a patio, a sculptor displaying artwork, naked women climbing fences, plazas, and natural scenery. Significant portions of imagery experienced by Subject 1 were novel (not from autobiographic memory) with integrated sensorium (except the taste sensation). For instance, Subject 1 described “There’s green pasture all around…oh my god I want to be there…I want to go there so bad…oh man so beautiful…I wish I was there man…I can feel the mist the green trees the fresh air oh my god man…god I wish I can be there.” In addition to the visual and auditory sensation, a mixture of other sensations was noted such as proprioception (heavy sensation, “I can’t move.”), vestibular (floating), temperature (cool mist), pains and visceral sensation such as “wanting to pee and a rush,” and pinching sensation of the stomach (See the Supplementary Tables). While describing such imagery, Subject 1 retained full awareness of reality as if he were a spectator within a virtual reality environment. Using a blindfold to cover the subject’s eyes enhanced the lucidity of the imagery. Subject 2 reported more positive responses with left sided stimulation in addition to 2–3 V right CeA stimulation. He reported a “genuinely good feeling,” “wanted to feel like this all the time,” and could cry from happiness.

Both subjects experienced negative responses. 4 V right dorsal BLA stimulation in Subject 2 provoked abdominal discomfort, salivation, anxiety, palpitations, chest tightness, and feelings of aggression like he “want[ed] to punch something.” 5 V stimulation caused him to feel “very pissed…not very good at all.” Twenty seconds later, he slammed his fists into the bed complaining of heat, thirst, and anger associated with feelings of disconnection similar to prior outbursts associated with his typical PTSD flashbacks.

Strong negative responses at moderate stimulation voltages were predominantly evoked contralaterally to the side that produced stronger positive responses. Thus, inverse coupling of emotional responses between amygdalae may exist.

Experiential phenomena (vivid mental imagery associated with intense feelings of familiarity and autobiographical components) were also elicited. For Subject 1, 2–3 V right HC stimulation evoked déjà vu and an out-of-body sensation. He also reported imagery of familiar scenes including childhood locales and prior travel destinations. For Subject 2, 3 V left dorsal BLA stimulation evoked déjà vu describing the sensations as “a subconscious feeling.” Both subjects experienced déjà vu ipsilateral to the side that produced euphoria.

Stimulation at 4–5 V evoked predominantly negative responses. Positive responses were elicited with 4 V stimulation of the left ventral BLA in Subject 1 and left CeA in Subject 2, however, 5 V stimulation of the same contacts elicited negative responses.

Hippocampus stimulation produced predominantly negative responses except with 1 V right HC stimulation in Subject 1 which elicited calming and cooling sensations that made him say “oh my god this feels so good.” 2 V right HC stimulation elicited a mixed response including pleasant déjà vu followed by increasing anxiety and twitching sensations.

Central nucleus stimulation in Subject 2 produced generally positive responses except at higher voltages. Right CeA stimulation in Subject 1 produced generally negative responses except at 2 V. The left CeA was not stimulated in Subject 1 due to preceding negative responses with left dorsal BLA stimulation.

Lastly, both subjects experienced pain or discomfort with various stimulation parameters. Subject 1 reported such sensations with 5 V left HC trial 1 (right upper arm pain), 2–3 V left ventral BLA trial 1 (stomach pain), 5 V left ventral BLA trial 2 (fear), and 4 V left dorsal BLA (chest tightness associated with lower extremity “heat stress” and nausea) stimulation, respectively. Subject 2 also reported uncomfortable sensations with 4 V left HC (chest tightness), 5 V left HC (right sided headache), 4 V left ventral BLA (stomach pain), 5 V left ventral BLA (throat discomfort), 4 V left dorsal BLA (stomach pain and intense anger), 4 V left CN (stomach pain), 5 V left CN (anxiety attack), 4 V right ventral BLA (left ear pain), 5 V right ventral BLA (intense generalized pain associated with PTSD), 4 V right dorsal BLA (stomach discomfort, hypersalivation, anxiety, tachycardia, anger), 4 V right CN (neck discomfort), and 5 V right CN (nausea and presyncope) stimulation, respectively.

Consensus results are displayed in Table 1. Refer to Supplementary Tables S1–S3 for verbatim transcriptions of subject responses to stimulation. Examples of the dreamy state and double consciousness are highlighted in Table 2.

**TABLE 1:** Acute stimulation effects of individual DBS electrode contacts in both subjects organized by contact stimulated and stimulation strength.

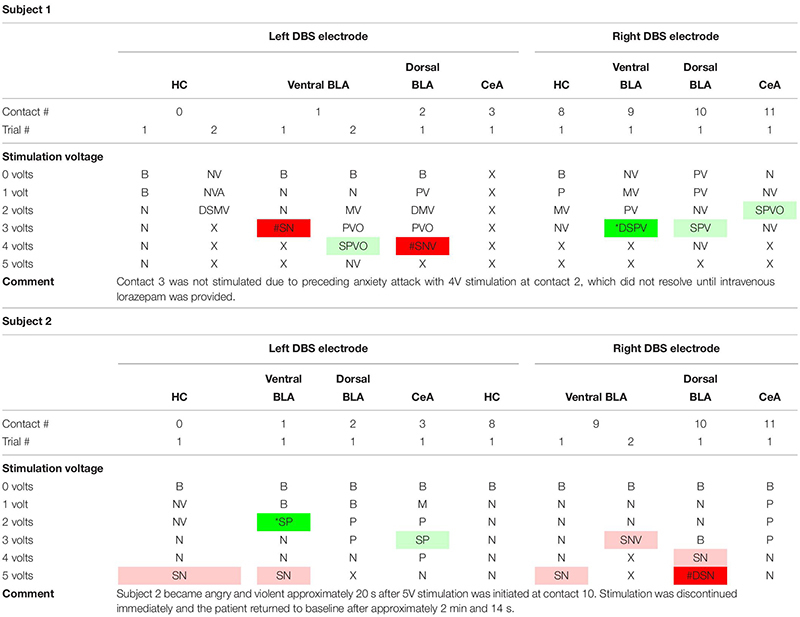

Subject 1 was stimulated with a frequency of 160 Hz and an initial pulse width of 120 μs for the first stimulation trial at contacts 0 and 1 for which he experienced significant negative responses. As such, all subsequent stimulation at other contacts and for repeat stimulation of contacts 0 and 1 was performed with a pulse width of 60 μs. Subject 2 was stimulated with a frequency of 160 Hz and a pulse width of 60 μs. HC, head of hippocampus; BLA, basolateral nucleus of the amygdala; CeA, central nucleus of the amygdala; P, mild to moderate positive responses including increased happiness, euphoria, relaxation, pleasure, and other comforting sensations; N, mild to moderate negative responses including increased anxiety, anger, pain, fatigue, fear, and other uncomfortable sensations; M, mixed response; S, strong response; B, baseline; D, delayed response; V, visual symptoms including experiential phenomena such as déjà vu or sensation of familiarity; O, olfactory symptoms; A, auditory symptoms; *, the most intensely positive response(s); #, the most intensely negative response(s); X, stimulation settings that were not tested due to safety concerns including avoidance of triggering PTSD, anxiety attacks, or further discomfort at higher stimulation strengths; red shading, strongest negative response(s); pink shading, stronger negative responses; green shading, strongest positive response; light green shading, stronger positive responses.

**TABLE 2:** Transcriptions of example responses from both subjects pertaining to the dreamy state and double consciousness.

**Structure**	**Voltage**	**Transcribed responses**
**Subject 1**		
Ventral BLA	2 V	“Now I’m at my elementary school…I’m at my elementary school… I’m like right outside the gate…[inaudible] ***…are they probing my brain? What a trip…it’s like I can’t get rid of this smile…and I’m right at the front gate where I went in elementary…why is it that I’m going back to where I grew up? What a trip…and that aqueduct I think I know where it’s at in ***…I just know where it’s at” (I) “Can you turn off the floating visualizations?” (S) “It just happens…yeah…like it’s changing a channel…it went from like the park to the aqueduct to the school…I float into it”
Ventral BLA	3 V	(S) “I’m in like a dark place now with a white wall… this one white wall like this and another one right here and I’m floating like right here…like two white walls perpendicular to each other” “This is not someplace you’ve been?” “Nuh uh…exactly I’ve never been here…like there’s grass…kind like I’m trapped a little bit…I feel like a rush in my upper body” “I’m over a driveway now…I’m over the driveway where I use to live in ***…oh my god…I’m over the driveway and there’s a big old oak tree a big ass oak tree the one that was in front of the house…I’m like to the side of it, the oak tree is right here…the house is behind it and the driveway is right here and I’m kinda of like floating right here…but what a trip I see the tree the oak tree and the driveway.” (I) And you’re smiling? (S) Yeah…I mean I don’t know it’s like I’m going back to a place I haven’t thought of in like shoot 30 years…I’m just thinking to myself why am I going back to *** places where I use to go when I was a little kid…what a trip” “I can do this all day…this is cool man” “If I ask you to can you visualize the room where you are right now, can you visualize me?” “Yeah I can visualize you and the bed, and me” “I’m floating over this place…but I think it was when I was in Germany, but I don’t really recognize it…and I’m just floating across it…what a trip man…is this what it feels like to be high?” “I think I’m in Germany…I think so yeah” “Can you voluntarily turn that off and just like focus” “yeah”
Dorsal BLA	1 V	(S) “Lady that is amazing…there’s this lady showing me all her ceramic artwork…she’s got all kinds of animals and she’s got this one it’s like a clownfish beautiful…I guess she’s like a sculpture…she does really good work…oh my god…all the animals that she made all the flowers they’re all glossy baby you got talent boy…I feel good…this lady she just showed me some nice artwork some nice things that she made…god she made 2 kettles one of the world and I don’t know what the other one is but one of the kettles is like the planet earth and she made it into a kettle…is that amazing or what…god…were like in a kind of [inaudible] place that’s been cleared out so the little houses and the lady the owner she does the ceramic artwork…beautiful…I see a penguin…oh man”
Dorsal BLA	3 V	(S) “I’m in the patio with a dog…it’s a cement patio…I think I’m in Costa Rica…yeah because the cement patio but the rest it’s all like forest…it’s like San Isidro…”
**Subject 2**		
Dorsal BLA	3 V	(S) “I am fine…oh yeah…I’m happy…almost seems like I had déjà vu…oh when you told you’d explain my eye…I just remember I had a dream about this…some of how do you say it…the tastes and the smells you know stuff like that” (I) “Déjà vu is new for you or it happens before too?” (S) “it’s only happened like twice before ever in my life?”
CeA	5 V	(S) “It’s like I want to…jolt…I just can’t it’s just a subconscious feeling …just run…just a subconscious feeling…”

(I) indicates interviewer’s queries and (S) represents subject responses. *** represents removal of potential patient identifiers.

## Discussion

Several intriguing findings were noted with the acute DBS of the amygdala in subjects with refractory PTSD: (1) Acute ES effects correlate well with the onset and offset of the stimulation. (2) Acute positive effects were associated with stimulation parameters in the mid-range voltage settings (2–3 V). (3) The positive effects seem to be inversely coupled with adverse responses from the same stimulation of the contralateral amygdala suggesting amygdala lateralization. (4) Adverse responses are predominantly noted at high-voltage stimulation (4–5 V). (5) Dreamy state and double consciousness were elicited during the ES in these two subjects without epilepsy.

The following discussions are based on the logic that our results do support Jackson’s concept of the “dreamy state” and “double consciousness,” which have been viewed as unique epilepsy-related phenomena. Our findings provide an essential control vs. the historical data that were obtained from subjects with epilepsy to suggest that the “dreamy state” and “double consciousness” might originate from an epilepsy-independent innate brain mechanism that could be activated by stimulation of the amygdala. The implications are discussed speculatively to incite neuroscientists’ interests in Jackson’s concept.

## The Dreamy State and Normal Dreaming

The term “dreamy state” was first coined by John Hughlings Jackson in 1888 as an “intellectual aura” of a “variety of epilepsy” that involves an “*elaborate or ‘voluminous’ mental state*” such as “’Reminiscence’; a feeling many people have had when apparently in good health” or a “’crude sensation’ (‘warning’) of (a) smell or (b) taste (or, when there is no taste, there may be movements, chewing, tasting, spitting)…or (c) the ‘epigastric’ or some other ‘systemic’ sensation” (Jackson, 1888; Taylor, 1931, 1958). He emphasized that the dreamy state, which involves reminiscence can occur without crude sensations and vice versa (Jackson, 1888). These experiential phenomena include vivid memories (déjà vu) involving intense autobiographical sensory hallucinations and illusions (Bancaud et al., 1994; Hogan and Kaiboriboon, 2003). In 1898–1899, based upon post-mortem pathological findings, he localized the dreamy state to the uncus within the temporal lobe calling them “uncinate group of fits” (Jackson and Colman, 1898; Jackson and Stewart, 1899).

Since then, other authors have also reproduced the dreamy state in patients with epilepsy utilizing ES of the temporal lobe, HC, and amygdala (Penfield and Jasper, 1954; Penfield and Perot, 1963; Halgren et al., 1978; Weinand et al., 1992; Vignal et al., 2007). Others have found that amygdala ES leads more easily to elicitation of the dreamy state (Gloor et al., 1982; Gloor, 1990). Bancaud reported 16 patients who experienced dreamy states with ES of medial temporal lobe structures (anterior hippocampus, amygdala, and temporal neocortex) and also described medial spread of discharges in more than half of dreamy states evoked by lateral temporal stimulation (Bancaud et al., 1994). A 2018 study found that déjà-rêvé, similar to the “reminiscence” of a dreamy state, could be further localized to the medial temporal lobe while the dreamy state as a whole (including crude sensations) was localized less specifically to the temporal lobe (Curot et al., 2018).

Jackson’s term, the “dreamy state,” is distinct from normal dreaming. Similar to Jackson, Vignal et al suggested that the “dreamy state” is based upon autobiographic memory (Vignal et al., 2007). In contrast, the “dreamy state” in our subjects encompassed not only autobiographic memory, but also novel not-previously experienced mental imagery. While dreamy states are accepted as symptoms of TLE, dreaming itself is a physiologic function. For our subjects, their experiences of vivid and emotional mental imagery in wakefulness secondary to amygdalae ES is similar both to Jackson’s “dreamy state” and physiologic dreaming in sleep lead one to speculate that both may utilize a similar neural network. Our results are unique in that epilepsy does not appear to be prerequisite for the elicitation of dreamy states. This is supported by findings that after-discharges are not necessarily required to produce dreamy states (Vignal et al., 2007). We only noted, among normal EEG, rare transient sharply contoured non-epileptic waveforms on scalp EEG recording over the right temporal lobe when euphoria was elicited with 3 V right ventral BLA stimulation in Subject 1.

Bancaud and Halgren have argued that, while amygdala ES has elicited dreamy states, results are often variable and it is difficult to consistently separate phenomena evoked by ES of the anterior hippocampus versus the amygdala (Halgren et al., 1978; Bancaud et al., 1994). Our study, however, allowed us to precisely stimulate specific portions of the amygdala at lower voltages due to our precise implantation of contacts within two portions of the BLA and CeA. Our testing showed that stimulation of the HC rarely elicited hallucinations or illusions while BLA stimulation did. We postulate that dreamy state generation likely involves a neural network encompassing the BLA and/or CeA of the amygdala.

Given the evidence suggesting that the dreamy state involves a neural network within the medial temporal lobe, we hypothesize that physiologic dreaming may also engage the same network. As early as 1883 there have been sporadic case reports including a more recent publication in 1984 by Murri, that reported patients with decreased levels or complete cessation of dreaming due to unilateral neoplastic or vascular cerebral lesions (Murri et al., 1984). These studies, however, did not explicitly localize lesions to specific neural structures such as the amygdala. More recent publications presented patients with Urbach-Wiethe disease who had bilateral medial temporal lobe lesions associated with decreased dream recall (Wiest and Brainin, 2014).

## “Double Consciousness”

John Hughlings Jackson also described a “double self” in association with dreamy states, during which actively seizing patients were simultaneously aware of a “double consciousness” including their own reality and another separate novel realm (Jackson, 1888; Taylor, 1931, 1958; Hogan and Kaiboriboon, 2003). For example, a woman with a dysplastic left amygdala experienced vivid autobiographical hallucinations with seizures during which she was “uncertain of the reality of her experience” and “not sure if (she) was having a seizure while dreaming, or only dreaming of (her) seizures” (Vercueil, 2005). Similarly, our subjects both reported being aware of reality while experiencing dreamy states with Subject 1 stating that the dynamically shifting mental imagery resembled switching television channels.

Our results support Jackson’s hypothesis that there are two coupled interlacing halves of human consciousness, which were described as the “object consciousness” versus the “subject consciousness” (Taylor, 1931, 1958; Hogan and Kaiboriboon, 2003; Vignal et al., 2007). In our study, we were able to electrically evoke a state similar to Jackson’s “double consciousness” in both subjects as they both maintained fully intact object consciousness whilst experiencing vivid dreamlike or reminiscent sensory phenomena (subject consciousness). In the future, if carefully designed questions can be asked during ES induced dreamy state and double consciousness, it may add further to the understanding of this aspect of human psychic function.

## The Amygdala as an Emotional Integrator for Dreaming

The neural circuitry of wakefulness and sleep is complex, but seems to involve various nuclei of the amygdala (Scammell et al., 2017). Identification of functional neuroanatomy for dreaming has remained as elusive as its physiologic function. The network responsible for physiologic dreaming may be larger than the medial temporal lobe region. In a contemporary publication by Hobson, he proposed a neural network responsible for dreaming which involves the amygdala, dorsolateral prefrontal cortex, basal ganglia, thalamic nuclei, inferior parietal cortex, cerebellum, visual association cortex, and even the primary motor and sensory cortices (Hobson and Pace-Schott, 2002). He additionally proposed the idea that the ascending arousal system first activates the forebrain before activating other structures such as the amygdala, which suggests that the brainstem is where dreaming processes are initiated (Hobson and Pace-Schott, 2002).

Within this network, we hypothesize that the amygdala functions as an emotional integration center for emotions in dreams, an idea that is supported by several lines of evidence. The amygdala determines the emotional load of dreams and may preferentially process fearful emotions due to its other function of determining emotional responses to stress (Adolphs et al., 1995; Schwartz and Maquet, 2002). Studies have shown that rapid eye movement (REM) sleep is associated with dreams (Nir and Tononi, 2010) and increased amygdala activation (Maquet et al., 1996; Nofzinger et al., 1997; Corsi-Cabrera et al., 2016). Two studies utilizing microstructural analyses and diffusion tensor imaging via MRI brain scans also found that the variations in microstructural integrity and volume of the amygdala affected dream duration, bizarreness, and vividness (De Gennaro et al., 2011, 2016).

Interestingly, our results also suggest that amygdala may regulate the bizarreness of dreams resembling symptoms like in “Alice in Wonderland Syndrome” (AIWS) such as distortions of various sensations and the sense of time (Fine, 2013). In our study, Subject 1 experienced unpleasant out-of-body experiences with 3 V right HC stimulation feeling distorted floating sensations associated with mental imagery of “big black glass walls going all the way up to the sky” with 1–3 V right ventral BLA stimulation. For Subject 2, 1 V left HC stimulation evoked brightened distortions of his left visual field associated with black silhouettes surrounding people and distant objects in the room. 2 V left HC stimulation evoked vertical lines like a box in space which distorted and obstructed half of his visual field. Cessation of stimulation lead to complete resolution of these symptoms. Unlike Subject 1, Subject 2 appeared to develop distorted illusory perceptions (metamorphopsias) rather than hallucinations which appeared to mimic sensory distortions seen in AIWS.

Our stimulation results support this idea that the amygdala and its subnuclei can generate vivid, emotional, bizarre, and dynamic mental sensorial imagery closely resembling physiologic dreaming suggesting that the amygdala may represent a final integration center of dream phenomena involving the five senses co-mingled with creative novel features and emotional elements.

## The Amygdala and Emotions

In a review of 64 publications involving human intracranial electrophysiology techniques, multiple locations of neural representation were identified for individual emotions which is supportive of emotions being generated by a wider neural network involving the amygdala (Guillory and Bujarski, 2014).

For both subjects, the strongest positive responses were elicited with ventral BLA stimulation. Additionally, when positive emotions were elicited by stimulating the ventral BLA, negative responses were evoked with contralateral ventral BLA stimulation. Similar to hemispheric lateralization of language, emotional valence may also be lateralizable (Lanteaume et al., 2007). In our subjects, lateralization of emotional valence did not relate to handedness as both subjects were right handed, but experienced euphoria with stimulation of opposite sides.

Interestingly, HC stimulation in our subjects led to predominantly negative responses bilaterally regardless of voltage strength. In contrast, CeA stimulation at moderate voltage levels (1–3 V) in Subject 2 elicited generally positive responses implying that the HC and CeA may also participate in the emotional network.

While euphoria has traditionally been difficult to localize, our findings suggest that the BLA and CeA may be important sites for eliciting euphoric responses. Other studies using intracranial ES of the temporal lobe, HC, and amygdala in patients with focal epilepsy have also reported ES-evoked orgasmic ecstasy (Surbeck et al., 2013; Chaton et al., 2018) whilst erotic feelings have been associated with mesial TLE as early as 1971 (Currier et al., 1971; Rémillard et al., 1983; Janszky et al., 2004). Additionally the anterior insular cortex has also been suggested to play a central role in the euphoria generation as evidenced by various functional studies of ecstatic seizures and a contemporary publication identifying up to 52 patients with seizures associated with intense positivity and even spirituality (Gschwind and Picard, 2016).

The inputs to and outputs from the amygdala include ventral amygdalofugal pathway, stria terminalis, basal forebrain, temporal lobe structures, olfactory stria, thalamus, hypothalamus, striatum, and brain stem (Whalen and Phelps, 2009). To investigate the neural circuits involved in the various stimulation conditions, we have done an analysis of the volume of tissue activated (Avecillas-Chasin et al., 2019). It showed that the positive effects arise from stimulating the basal nucleus and associate with the ventral amydalofugal pathway. The negative effects mainly result from the lateral nucleus and the hippocampal area, which also associate with stria terminalis.

The amygdala has also been thought to be involved in the experience of pain. Similar to the emotions, pain is processed via a broader neural network involving the amygdala and its subnuclei (Veinante et al., 2013; Neugebauer, 2015; Thompson and Neugebauer, 2017). Evidence suggests that pain may trigger BLA hyperactivity leading to feedforward inhibition of the medial prefrontal cortex resulting in cognitive dysfunction, uninhibited BLA hyperactivity, loss of CeA inhibition, and pain perseverance (Ji et al., 2010; Neugebauer, 2015; Thompson and Neugebauer, 2017). Both of our subjects experienced discomfort at higher voltage strengths, suggesting that BLA and CeA hyperactivity may be induced by higher voltage stimulation and should therefore be avoided when selecting therapeutic parameters. Interestingly, the CeA appears to also play a major role in pain suppression during extremely stressful situations (Neugebauer et al., 2004; Veinante et al., 2013) and may be targeted for ES therapy to induce hypoalgesia, improve cognition, and even elicit euphoria.

## Creativity

Lastly, we briefly discuss human creativity for which there is limited literature regarding its cerebral localization (Baas et al., 2015). Both of our subjects experienced novel vivid mental imagery that was not previously experienced. Subject 1 saw unfamiliar people, animals, locations, cars, buildings, scenery, walls, sculptures, artwork, faces, demons, and objects including dirty mattresses. For Subject 2, stimulation of the left HC evoked unfamiliar visual distortions including silhouetted objects, left visual field brightening, and vertical lines within a box. As such, ES of the amygdala appears to also generate dynamic and evolving unique mental imagery, which may represent a creative function and suggests that the amygdala and medial temporal lobe may be involved in the network responsible for human creativity. This anatomical association of the medial temporal lobe with generation of novel creative imagery is striking given reported cases of late-life *de novo* artistic creativity following injury of the temporal lobe, most commonly seen in fronto-temporal dementia (Miller et al., 1998; Pollak et al., 2007).

## Concluding Remarks

Stimulation of the amygdala with DBS electrodes in awake patients presents unique opportunities to evaluate DBS for PTSD treatment and to examine stimulation effects independent of depth electrode stimulation in refractory TLE which has dominated the literature for ES of the amygdala. Our results indicate that the dreamy state can be evoked by ES of the amygdala in awake patients without epilepsy, euphoria can be evoked with moderate ES of the BLA and CeA, emotional valence evoked by ES may exhibit hemispheric dominance irrespective of handedness, and high voltage stimulation strengths should probably be avoided due to significant negative responses in both subjects. Since behavioral effects of ES of the amygdala appear to vary individually and subjects can experience discomfort or negative responses at certain stimulation parameters, we recommend that, prior to initiation of chronic stimulation, every subject undergo mapping of acute behavioral responses to ES in a monitored setting with simultaneous continuous VEEG monitoring supervised by a qualified team. The results of the acute mapping may be utilized to guide optimal selection of therapeutic stimulation parameters during chronic DBS of amygdala.

It is beyond the scope of this manuscript to discuss the mechanistic control of the dreamy state and double consciousness adequately. In short, we speculate that the amygdala could act as a television channel selector, as experienced by Subject 1 during the stimulation. This process probably requires fine-tuning of the attention network of the brain, which is shown to connect with the amygdala (Zikopoulos and Barbas, 2012).

Lastly, why we dream is an unanswered question as old as human history. We hypothesize that dreaming and human creativity are interconnected as innate cerebral mechanics, which are structurally and functionally linked to the anterior mesial temporal lobe, especially the amygdala and its interconnected networks. In the elicited dreamy state, a person becomes like a spectator in a virtual realm that is dynamic, unsolidified, emotional, and dramatic, consisting of autobiographic memories intermixed with novel imagery, strange characters, and unique scenarios. The generation of novel and not-previously experienced unique imagery via ES of the amygdala is especially intriguing as it suggests that the human brain may rely on the same neural network to generate both dream and creativity related mental imagery, thoughts, and concepts. This shared network may support the idea that dreaming in sleep might enrich the creativity of insight and responses to daytime challenges (Barrett, 2017; Llewellyn and Desseilles, 2017). If so, this neural network involving the amygdala as a crucial node is evidently vital for the continuing survival and development of individuals, societies, and the human race as a whole.

## Limitations

Our report is limited to two subjects without healthy controls, which limits generalizability. We prioritized avoiding significant adverse responses; therefore, Subject 1 was aware of certain parameter changes allowing for premature stimulation termination while avoided testing certain contacts. Despite good correlation of effects between the on and off of stimulation, Subject 1 also had occasional prolonged responses to stimulation and experienced delayed return to baseline for a few seconds between contacts at times. Additionally, our results are limited to our specified stimulation parameters and DBS electrodes. Future studies are needed to introduce more formal protocols, monitor additional parameters, maintain full blinding, and focus on the reproduction of responses.

## Data Availability Statement

All datasets generated for this study are included in the article/Supplementary Material.

## Ethics Statement

This study was carried out in accordance with the recommendations of IRB of the Greater Los Angeles Veterans Affairs Healthcare System with written informed consent from both subjects. Both subjects gave written informed consent in accordance with the Declaration of Helsinki. The protocol was approved by the IRB of the Greater Los Angeles Veterans Affairs Healthcare System.

## Author Contributions

J-PL and RK were responsible for obtaining institutional review board (IRB) approval, completing the Food and Drug Administration Investigational Device Exemption (FDA IDE) application, and obtaining informed consent from each subject. J-PL was responsible for neurosurgical implantation of the DBS device and electrodes. JC, J-PL, SK, and RK were responsible for data collection during the acute stimulation procedure. GL and JC were responsible for data review, independently reviewing video electroencephalogram recordings, transcribing each subject’s spoken responses, and writing the manuscript. GL, JC, J-PL, RK, SK, and AB were responsible for contributing to manuscript revision and providing final approval for the submitted version. J-PL and AB contributed neuroimaging figures utilized in the manuscript.

## Conflict of Interest

The authors declare that the research was conducted in the absence of any commercial or financial relationships that could be construed as a potential conflict of interest.
