# Supplementary material for: Acute Effects and the Dreamy State Evoked by Deep Brain Electrical Stimulation of the Amygdala: Associations of the Amygdala in Human Dreaming, Consciousness, Emotions, and Creativity
Source: Front Hum Neurosci. 2020 Feb 25;14:61. doi: 10.3389/fnhum.2020.00061 (PMC7052301; doi:10.3389/fnhum.2020.00061)
Supplement: Supplementary file 1 [file Table_1.docx]

# Supplementary Material

**Table S1.** Transcriptions of responses from both subjects pertaining to the dreamy state with autobiographic memory recollection of experiential phenomena. (I) indicates interviewer’s queries and (S) represents subject responses. *** represents redaction of potential patient identifiers.

| Subject 1 | | |
| --- | --- | --- |
| Structure | Voltage | Transcribed Responses |
| Right HC | 2V | (S) “I got déjà vu now…that I was here before and doing the same exact thing…oh my god…I feel like I was here right now with you and him and everything…oh my god…it’s a trip because I feel like I was here before…it’s like indifferent…I feel like a little anxious right here…more like in my chest…”  (S) “Ah man that’s a rush…again I feel like that cool rush…it comes from top of my brain like cool water just coming down…from here to here…felt good…only thing that remains is that my muscles want to twitch...it’s weird you know it’s a weird feeling |
| Right HC | 3V | (S) “What a trip…I feel kind of like out of my body…just kinda like floating around…seeing what is going around with the wires” |
| Right Ventral BLA | 1V | (S) “I’m in somebody’s garage…it’s got the garage door open and there’s a bunch of junk there and I’m just hovering…”  (S) “Um…this sounds ridiculous but it looks like I’m floating by this park in *** that I use to go there as a kid…just outside the *** yeah”  (I) “Is it a pleasant feeling to be there?”  (S) “It’s a trip cause it looks like I’m floating and I’m seeing it and I haven’t thought of it in like so many many many many years”  (I) “You can actually visualize it?”  (S) “Yeah…a rush in my whole body…big time rush…rush now in my head its inside my head” |
| Right Ventral BLA | 2V | (S) “Now I’m at my elementary school…I’m at my elementary school… I’m like right outside the gate…[inaudible] ***…are they probing my brain? What a trip…it’s like I can’t get rid of this smile…and I’m right at the front gate where I went in elementary…why is it that I’m going back to where I grew up? What a trip…and that aqueduct I think I know where it’s at in ***…I just know where it’s at” |
| Right Ventral BLA | 3V | (S) “I’m over a driveway now…I’m over the driveway where I use to live in ***…oh my god...I’m over the driveway and there’s a big old oak tree a big ass oak tree the one that was in front of the house…I’m like to the side of it, the oak tree is right here…the house is behind it and the driveway is right here and I’m kinda of like floating right here…but what a trip I see the tree the oak tree and the driveway”  (I) “And your smiling?”  (S) “Yeah...I mean I don’t know it’s like I’m going back to a place I haven’t thought of in like shoot 30 years…I’m just thinking to myself why am I going back to *** places where I use to go when I was a little kid…what a trip”  (S) “I can do this all day…this is cool man”  (S) “I don’t know if it is Germany, but it’s that place where I think it’s Germany and this time I’m just floating I’m not moving I’m just floating there” |
| Right Dorsal BLA | 3V | (S) “I’m in the patio with a dog…it’s a cement patio…I think I’m in Costa Rica…yeah because the cement patio but the rest it’s all like forest…it’s like San Isidro…” |
|  | | |
| Subject 2 | | |
| Structure | Voltage | Transcribed Responses |
| Left Dorsal BLA | 3V | (S) “I’m fine…oh yeah…I’m happy…almost seems like I had déjà vu…oh when you told you’d explain my eye…I just remember I had a dream about this…some of how do you say it…the tastes and the smells you know stuff like that”  (I) “Déjà vu is new for you or it happens before too?”  (S) “It’s only happened like twice before ever in my life.” |
| Right Ventral BLA | 5V | (S) “It feels like a lot of my symptoms are being brought up from when I first got here…I guess and the physical, the pain, not pain, but taste, the tachycardia…yeah its happening right now…I’m remembering all the stuff when I first got injured…right now I got the stomach I can feel the pinching…I can feel that…the numbness in my mouth, taste…the pain in my wrist…I feel nauseous”  (S) “Yeah I don’t feel good at all…this is making me remember when I got blown up…just the pain…my body is going through it so…yeah right now like I wanna blackout” |

**Table S2** Transcriptions of responses from both subjects pertaining to euphoric sensations. (I) indicates interviewer’s queries and (S) represents subject responses. *** represents redaction of potential patient identifiers.

| Subject 1 | | |
| --- | --- | --- |
| Structure | Voltage | Transcribed Responses |
| Right Ventral BLA | 3V | (S) “I’m over a driveway now…I’m over the driveway where I use to live in ***…oh my god...I’m over the driveway and there’s a big old oak tree a big ass oak tree the one that was in front of the house…I’m like to the side of it, the oak tree is right here…the house is behind it and the driveway is right here and I’m kinda of like floating right here…but what a trip I see the tree the oak tree and the driveway.”  (I) “And your smiling?”  (S) “Yeah...I mean I don’t know it’s like I’m going back to a place I haven’t thought of in like shoot 30 years…I’m just thinking to myself why am I going back to *** places where I use to go when I was a little kid…what a trip.”  (S) “I can do this all day…this is cool man.”  (S) “I’m floating over this place…but I think it was when I was in Germany but I don’t really recognize it…and I’m just floating across it…what a trip man…is this what it feels like to be high?”  (I) “It seems like your mood is pretty good.”  (S) “Yeah…this is something else…what a trip.” |
|  | | |
| Subject 2 | | |
| Structure | Voltage | Transcribed Responses |
| Left Ventral BLA | 2V | (S) “Emotionally now I’m fine…a little bit of tightness in the muscles other than that no…mostly in my hip my left hip neck and right temple…other than that, no I feel good…I think bought stuff right, I feel some euphoria…yeah…I’m happy”  (I) “Is that a different kind of happiness than you experience on a day to day basis?”  (S) “No I mean well… no I mean this is the happiest I’ve been since we started turning it on…no prior to that I just like joking around and having fun”  (I) “I want to ask you one more time about the euphoric sensation. What was that last time you naturally experienced something like this?”  (S) “That’s a good question…I can’t even remember the last time I’ve had a happy thought”  (I) “Not like this? This is unique?”  (S) “Yeah…this one is better than the rest…cause I mean…my daughter you know makes me happy so but without just- without her and just being by myself that’s the first time…so…uh this is a different feeling…cause I didn’t have anybody else…uh to make me think happy thoughts”  (I) “Would you say that this is stronger more intense than the joy that your daughter brought you?”  (S) “Yeah I would…yeah…I mean the happiness I felt yeah…it was a lot more…it actually made me feel normal for the first time...yeah…its interesting for me” |

**Table S3.** Transcriptions of responses from both subjects pertaining to the novel experiences in the dreamy state including symptoms of visual distortion similar to those reported in Alice in Wonderland Syndrome. (I) indicates interviewer’s queries and (S) represents subject responses. *** represents redaction of potential patient identifiers.

| Subject 1 | | |
| --- | --- | --- |
| Structure | Voltage | Transcribed Responses |
| Left HC  Trial 2 | 1V | (S) “I’m seeing some ugly faces…I’m seeing some ugly faces”  (S) “I feel like I’m heavy…I feel like I’m heavy…and I’m by myself…I’m by myself in the garage and I feel like I’m heavy…I can’t move…there’s a car a black car trying to make a right turn but it can’t it’s too narrow so I guess he backed up…it’s too thin you know so the car couldn’t make that turn so he took off”  (S) “I don’t feel good”  (S) “I’m having these little things popping around me…there popping around me these little things are…there like little plastic bags and they’re popping...they’re popping…yeah like those little plastic bags when squeeze them they pop…that’s what I’m hearing that’s what I’m seeing but nobody’s doing it they’re just doing it on their own” |
| Left HC  Trial 2 | 2V | (S) “That feels good…that feels good whatever you’re doing it feels good…oh god that feels good…keep me there please”  (S) “That feels so good leave me here please…I feel this cool breeze oh my god…but I see a street a dirty street…I still feel a cool mist but I see a dirty street a car passing…and on the other side of the street it’s all country it’s like a big old valley…if I wasn’t so heavy I could just walk over across the street and go to it…but I can just go to the valley it feels good but I can’t but I’m [inaudible] garage…I guess I’m obese I can’t move…yeah and it’s just like maybe 50 meters to cross that fence…aw man…”  (S) “Alright [inaudible] that feels good…ah that feels good boy…keep pushing that button it feels so good”  (S) “I got tightness chest now…I got tightness of chest”  (S) “I feel like someone’s mad at me and I see some faces…they’re mad at me I didn’t do anything” |
| Left Ventral BLA  Trial 2 | 2V | (S) “I keep lingering at this like apartment complex…it’s the same place I was at earlier but now I’m at a different place why do I keep lingering there... I see kids playing …but why do I keep lingering...I see this lady come out too with her bags”  (S) “Just a little bit tingling in my legs that’s it…yeah tingling”  (S) “I have a positive feeling coming right now but it’s not that cool feeling but it’s okay…I’m next to a couple of big old oak tress…big old twitch in my chest”  (S) “I keep having like these twitches in my chest” |
| Left Ventral BLA  Trial 2 | 3V | (S) “I feel like I want a chocolate chip cookie now…I want a chocolate chip cookie…man I can smell it and I want one…what a trip…and I can see like this small kitchen there’s a stove right there…its outside though…it feels good its outside I smell cookies”  (S) “I feel okay you know I’m outside and that lady’s cooking cookies and I feel okay…” |
| Left Ventral BLA  Trial 2 | 4V | (S) “There’s green pasture all around…oh my god I want to be there…I want to go there so bad…oh man so beautiful…I wish I was there man…I can feel the mist the green trees the fresh air oh my god man…god I wish I can be there”  (S) “They have little round windows so people can I guess look in or they can look out…it’s kind of like in the rainforest but it’s a little house…so peaceful right there…physically I feel okay…I just want to go there…god that’s so beautiful it’s just a little house and the whole valley of green just surrounding it oh my god I can smell the mist I can feel the mist”  (S) “God I wish I was there man…leave me here would you…I want to stay here…god it’s so beautiful it’s so green it’s so mist… temperature is perfect man…aw don’t get me away from here don’t take me away” |
| Left Ventral BLA  Trial 2 | 5V | (S) “Not so good now I’m like in a little town and I’m carrying like a piece of wood…aw man…I’m seeing um images ugly images he’s mad he’s pissed to why is everyone pissed why is everyone mad at me?”  (S) “It’s like I’m not welcome there I can’t go there I’m not welcome there” |
| Left Dorsal BLA  Trial 1 | 1V | (S) “Lady that is amazing…there’s this lady showing me all her ceramic artwork…she’s got all kinds of animals and she’s got this one it’s like a clownfish beautiful…I guess she’s like a sculpture…she does really good work...oh my god…all the animals that she made all the flowers they’re all glossy baby you got talent boy…I feel good…this lady she just showed me some nice artwork some nice things that she made…god she made 2 kettles one of the world and I don’t know what the other one is but one of the kettles is like the planet earth and she made it into a kettle…is that amazing or what…god…were like in a kind of [inaudible] place that’s been cleared out so the little houses and the lady the owner she does the ceramic artwork…beautiful…I see a penguin…oh man” |
| Left Dorsal BLA  Trial 1 | 2V | (S) “I’m having twitches…I don’t like the way it feels because its giving me images of ugly faces…and I feel cloudy…I feel cloudy…mentally I feel cloudy…I’m seeing these ugly faces…one of them is a biker…he’s trying to kick down the wall...why do I keep having walls…it’s like every place I go there’s a wall…he’s trying to kick it down” |
| Left Dorsal BLA  Trial 1 | 3V | (S) “Okay now I’m feeling a lot better…I’m sitting down on top of that wall and looking that way…[inaudible]…I don’t know how I got up there but I’m really comfortable I’m sitting down looking around and he’s still trying to get up…don’t ask me how I got up here cause I got no idea…beautiful…oh man I can just feel the mist boy…I can see this beautiful green valley with the trees and the grass…leave me here man…and I can feel like the moist air just breathing it”  (S) “Yes I’m okay…I’m just really good feeling I’m just peaceful right now…I’m laying down next to the house, there’s metal beams but it still feels good…feels very good…this is the spot right here whatever you’re doing this is it god I not only do I feel comfortable but I feel cool mist all around me…there’s that lady again but she’s not mad anymore…she’s right there that lady with the blue dress…god she’s not mad…I see pizza boxes a bunch of pizza boxes and I don’t know if the lady is the owner of the pizza places but I’m not going to make pizzas…she came down from where she was up and I’m with her right now and she’s trying to tell me to help her with the pizzas and I’m like no…I’m not getting near that oven…now she’s mad at me…no she’s not…she not mad at me she put the pizzas like in this other little oven that’s what she was doing she’s not mad at me…god…so I put the pizza in the oven she’s not mad at me anymore but she took off” |
| Right Ventral BLA | 1V | (S) “I’m in somebody’s garage…it’s got the garage door open and there’s a bunch of junk there and I’m just hovering…” |
| Right Ventral BLA | 3V | (S) “I’m in like a dark place now with a white wall… this one white wall like this and another one right here and I’m floating like right here…like 2 white walls perpendicular to each other”  (I) “This is not someplace you’ve been?”  (S) “Nuh uh…exactly I’ve never been here…like there’s grass…kind like I’m trapped a little bit…I feel like a rush in my upper body” |
| Right Dorsal BLA | 1V | (S) “I feel okay…I’m back at an apartment complex…I don’t recognize it…why am I going back to these places?...I’m still at that apartment complex…it’s like a condominium complex actually…I’m think I’m just a little to the side where I was floating early where I said there was an open garage…I’m to the right of that like 2 garages down but all the garages are closed and I’m on the street.  (I) “You still have a smile on your face”  (S) “It’s a trip man”  (S) “A little [inaudible] head rush…”  (S) “Yeah I don’t feel good…just like real anxious…and tightness in chest…”  (S) “I feel cooling sensation…not as good as earlier…but I feel it…yeah…this is a trip”  (S) “I’m just enjoying the ride…why not”  (S) “I feel a little bit of tension in my shoulders and upper chest”  (S) “I see 2 really dirty mattresses…oh man they are really dirty…they’re blue mattresses and they’re on the street…they have big stains on them…get out of here man…”  (S) “I see some girls…they’re like swinging…they’re like playing like in a little yard area…4 girls…”  (S) “Ok I’m like oh man I think I’m back in Germany…yeah it’s all green…god that’s beautiful man…I feel like I just want to grab a tree…I want to grab a tree just hug them...God that’s beautiful…like green pastures, trees, mountain behind them, blue sky” |
| Right Dorsal BLA | 2V | (S) “Big black glass going all the way to the sky and I’m looking up at it…it’s like big black glass and it goes up all the way to the sky and I’m looking at it…it feels like I’m trapped I can’t [inaudible] it’s so high …I want to go above it but I can’t”  (I) “Any other sensations in your body?”  (S) “Wanting to pee and a rush”  (S) “This glass wall is huge man” |
| Right Dorsal BLA | 3V | (S) “I’m in the patio with a dog…it’s a cement patio…I think I’m in Costa Rica…yeah because the cement patio but the rest it’s all like forest…it’s like San Isidro…well again I see some kids I see some adults…she’s doing laundry…she’s doing laundry in a bucket…this lady is doing laundry…she’s pissed…she’s [inaudible]…I don’t know she’s doing laundry in a bucket…she’s wearing blue dress and shoes…she’s pissed she’s mad”  (I) “Feeling anything else in your body and new sensations?”  (S) “A little happiness…yeah…I’m happy because that’s lady mad at me…I didn’t do anything to her…she’s just pissed…gotta get her a washing machine”  (I) “Are you feeling any anxiety?”  (S) “Um…yeah a little bit…good anxiety though”  (S) “She’s not in my field of view anymore”  (I) “Do you still feel happy?”  (S) “Yeah” |
| Right Dorsal BLA | 4V | (S) “Pee sensation is still there big time…okay I’m feeling like a cold rush it doesn’t feel good at all…it doesn’t feel good…no…whatever he’s doing it doesn’t feel good…causing a rush in my legs…a cold rush…I don’t like it”  (S) “It’s a hot rush that doesn’t feel good…my stomach feels like anxious…and I see some…I’m in a place where there’s some plywood on that side…someone nailed them on that side…and I’m over here…why do I keep coming across wall? …that’s like the third wall already…the great china wall…the big glass wall and now this plywood wall…wall am I coming across these walls?...”I don’t understand why I keep coming why these images of walls keep coming to me…the great china wall was pleasurable but these 2 are so so…the last one wasn’t good at all…there’s like a rush a hot rush in my legs…it looks like its fading away but it’s still there” |
| Right CeA | 1V | (S) “I’m in an underground garage under a building and somebody a car pushed over a statue a white statue… there’s no other people out there it’s just me and them and he ran into a statue and the statue fell down”  (I) “How do you feel?”  (S) “Nervous…yeah…I don’t like the way I feel right now…I feel like tightness in my forehead…I don’t like this feeling at all” |
| Right CeA | 2V | (S) “I feel a little bit of calm now…and I smell bread…I’m coming down like this staircase…like a wooden garden ladder…and I’m not on it I’m just in front of It and I smell bread…and its outside too and it’s all green …it’s all green and there’s {inaudible] flowers…god that smells good man…yeah it’s like fresh bread I can see that loaf of bread...it’s a white loaf of bread this big…god…and it’s a beautiful place its actually cool…ah man I feel this cool sensation right here”  (S) “God I feel twitches but it’s perfect…I’m like sitting down in a garden chair and just enjoying the coolness…and the mountains right here and the mountain and it’s green and there’s a valley a big old valley right there…ah man and I’m sitting down in a chair like a garden chair the small ones…I’m just enjoying it oh my god…so beautiful”  (S) “It’s so cool I can feel like the mist of the clouds too…ah man that feels good boy”  (I) “Do you feel calm?”  (S) “I did”  (I) “Tell me your anxiety right now”  (S) “This is weird…my anxiety went up a lot…yeah and I got this real bad tightness in my chest…I don’t like this I don’t like the feeling whatever you’re doing I don’t like it” (S) “Yeah I have a nice cool breeze in the chest and a… calmness…yeah this is it right here man…don’t change it leave it right here…I’m in like a green forest and its nice a cool and there’s a tree right in front of me and there’s trees all around me…oh yeah that feels good”  (S) “I’m in front of…there’s like an entrance gate I’m in front of it now…I guess I’m floating now but I’m in front of that entrance gate to that place where I was at and it’s like cement but it’s really beautiful it’s like a gateway into the back where everything is green…ah it feels good…that feels so good whatever you’re doing” |
| Right CeA | 3V | (S) “Tightness of chest…yeah there’s like a…I’m like between 2 houses and there’s a white gate right there a white gate and I’m in this property and there’s a different property on the right side of me…again I’m just like trapped…and I’m seeing ugly images too…I’m seeing ugly images…distorted fox, evil faces with eyes and ears, they are very clear…” |
|  | | |
| Subject 2 | | |
| Structure | Voltage | Transcribed Responses |
| Left CeA | 1V | (S) “I see it brighter on this side…yeah when I look I can look to the left like when I scan everything kind of has a shadow like a light bright shadow…how do you say it…it looks different…everyone has a halo a black silhouette around them…confused…honestly I can’t figure out why I’m seeing this…my stress level is up a little bit…when I look at you guys your white jacket white coat got a grey shadow around it same thing with his shirt”  (S) “Just the colors of the…it’s like a bright light flicking up and down and the shadows around it” |
| Left CeA | 2V | (S) “The room just seems brighter is all…more light…it’s like you guys are adjusting my eyes like adjusting the color of the TV…right now my anxiety is up a little bit…so I got a little bit of tachycardia cause I know I’m anxious I’m confused why I’m seeing this way and it kind of bothers me that I’m seeing this way…my throat is kinda tight and chocking…yeah only thing that is confusing is that the lines on the wall and the shadows around you” |
| Left CeA | 5V | (S) “Anxiety...just a little anxious…it’s like I want to…jolt…I just can’t it’s just a subconscious feeling …just run…just a subconscious feeling…my chest is increasing tension right side of my brain feels like it’s hurting…headache…and my eyes are focusing on stuff that seems brighter…pretty much my body is trying to tell me to get it to stop” |
